# Rescue Operations Lead to Increased Cardiovascular Stress in HEMS Crew Members: A Prospective Pilot Study of a German HEMS Cohort

**DOI:** 10.3390/jcm10081602

**Published:** 2021-04-09

**Authors:** Markus Strauss, Janosch Dahmen, Sophia Hutter, Marko Brade, Roman Leischik

**Affiliations:** 1 Department of Cardiology I—Coronary and Peripheral Vascular Disease, Heart Failure Medicine, University Hospital Muenster, Cardiol, 48149 Muenster, Germany; 2 Department of Cardiology, Faculty of Health, School of Medicine, University Witten/Herdecke, 58095 Hagen, Germany; sophia.hutter@uni-wh.de; 3 Department of Trauma and Orthopedic Surgery, Faculty of Health, School of Medicine, University Witten/Herdecke, 51109 Cologne, Germany; janosch.dahmen@uni-wh.de; 4 Berlin Fire and Emergency Medical Service, Medical Director Committee, 10179 Berlin, Germany; 5 BG Klinikum Duisburg, Department of Anesthesia and Intensive Care, 47249 Duisburg, Germany; marko.brade@bg-klinikum-duisburg.de

**Keywords:** helicopter emergency medical service, HEMS, cardiovascular stress, emergency physician, paramedic

## Abstract

Helicopter emergency medical service (HEMS) is an essential part of prehospital emergency medicine. The working conditions lead to high physical stress, especially in rescue operations. The study aimed to determine the cardiovascular stress profile during rescue situations in HEMS crew members. Twenty-one HEMS crew members (male *n* = 20) participated in the prospective study. Heart rate, blood pressure and long-term ECG measurements were recorded during the whole operation day. The changes of measurements during rescue operation (52 operations in total) were compared to these of standby time. Rescue operations lead to increased load on the cardiovascular system, as expressed by significantly higher blood pressure, heart rate values and rate of cardiac events compared to standby time. Of special note, the difference in systolic blood pressure mean was 7.4 ± 9.0 mmHg (CI [5.1; 9.7], *p* < 0.001). Maximal heart rate was on average 33.7 bpm higher during rescue operation than in the standby time (CI [26.2; 40.8], *p* < 0.001). Cardiac events occurred significantly more frequently during the period of rescue operation than in standby time hours (*p* = 0.02). The results reported a significant load on the cardiovascular system during rescue operations in HEMS crew members. Therefore, it is necessary to carry out a risk stratification of the HEMS crew members to prevent cardiovascular risk and events.

## 1. Introduction

The working conditions in the helicopter emergency medical service (HEMS) are characterized by high physical and psychological stress in rescue situations [1]. Previous studies in professional groups of public services—in particular in the fire and police departments—have shown a significant cardiovascular risk in these groups [2]. In firefighters, sudden cardiac death is the leading cause of death on duty [3]. Helicopter emergency medical service is an important pillar of security in the field of rescue services [4]. It has become an integral part of increasing importance in prehospital emergency medicine [5]. To date, however, there are only a few scientific studies that have quantified physical stress, in particular cardiovascular stress, in the HEMS crew in action. A study by Petrowski et al. [6] demonstrated a significantly higher cardiovascular burden in the air rescue service than in the hospital’s clinical activity. Benzer et al. [7] analyzed the physiological cardiovascular strain among HEMS crew members in 1991. For this purpose, cardiovascular load was examined at different times during use (alarm notification, approach, landing, outbound flight to the hospital, hospital handover, return flight). The researchers were able to demonstrate a significantly higher cardiovascular load in use than in the idle time. Recent studies by Carchietti et al. [8], who examined the change in heart rate (HR) during rescue operations, were able to demonstrate a relevant cardiovascular strain, particularly in complicated and longer-lasting operations. A decisive role in this seems to be played by dispatch- and mission-related stress of the autonomic nervous system [6].

Overall, the cardiovascular load of HEMS crew members has been insufficiently investigated. Therefore, the aim of this study was to determine the cardiovascular stress profile during rescue operations and to compare these results with the cardiovascular profile during standby time.

## 2. Materials and Methods

### 2.1. Study Participants

The study participants were active as medical staff at a German rescue helicopter base. A total of 21 subjects (male *n* = 20) participated in the study (11 emergency physicians and 10 paramedics). The investigation included at least one full day of work and started at 7:00 a.m. until the end of duty at sunset. A total of 52 emergency operations were evaluated. The examination protocol was approved by the Central Ethics Commission of the University of Witten/Herdecke (No. 35/2016), and the examination was carried out according to the rules of the Helsinki Declaration. We obtained written, informed consent from all participants.

### 2.2. Study Design and Procedure

This study was conceived as a prospective pilot study to examine the cardiovascular load of HEMS crew members during rescue situations. For this purpose, the blood pressure (BP) and HR profile, as well as cardiac arrhythmia in use, were compared with those of the resting phases between rescue situations (on the same day). The investigations took place from November 2016 to September 2017. For this purpose, a long-term BP and long-term ECG were set up for the emergency physician on duty and the paramedics for the entire day at work. Before going on duty, height, weight, body mass index (BMI) and abdominal circumference were recorded. Body height was measured using a commercially available stadiometer. A digital bathroom scale from the manufacturer Smart Weigh, model SBS500, was used to determine body weight. BMI was determined according to the following formula: body weight/(height in m)^2^. The abdominal circumference was measured in a standing position at the end of expiration, in a horizontal plane, midway between the lower edge of the rib and the upper edge of the iliac crest [9]. The European Society of Cardiology (ESC) risk score was used to calculate the cardiovascular risk scores of the participants [10]. Specifically, in our investigations, we applied the 10-year cardiovascular risk score calculator.

### 2.3. Long-Term Blood Pressure and Long-Term Electrocardiography Measurements

For long-term blood pressure (BP) measurements (ABPM) and 24-hour Holter ECG electrocardiography (ECG) we used ABPM devices from Custo GmbH (Cusot screen 400). The device saves synchronous recordings of long-term BP and long-term ECG measurements and ECG signal is accepted via the Custo guard 3 and Custo belt 3 by radio. We installed the devices at the start of the service. This technique included a BP cuff for non-invasive BP measurement and an elastic 3-channel electrode belt with ECG transmitter (Custo belt and Custo guard). The Custo screen 400 Holter ABPM recorder was attached to the subject’s belt with a designated pocket. The long-term ECG recorded continuously throughout the working day. In the beginning, a resting measurement was made after 5 min of physical relaxation. The device for ABPM measurement started with a manual measurement at the push of a button. The BP measurement intervals were set as 10-min intervals, both in the non-operational time and during the operation. In the event of incorrect measurements, an immediate automatic re-measurement was programmed to be carried out.

BP and HR were measured continuously throughout the day. The examination time was divided into “rescue operation” time (including the time from the receipt of the alarm message to the clinical patient transfer) and “standby time” (consists of all values between operations). The systolic BP and diastolic BP were evaluated according to the minimum (BP dia min and BP sys min), maximum (BP dia max and BP sys max) and mean (BP dia mean and BP sys mean) values with standard deviation in standby time and during rescue operation time.

The HR was evaluated simultaneously with the BP according to the minimum (HR min), maximum (HR max) and mean (HR mean) values with standard deviation during standby time and rescue operation time. We recorded the number of occurrences of extrasystoles (supraventricular, ventricular). These were compared to their occurrence in rescue operation vs standby time. We interpreted the increases in BP, HR and extrasystoles as an expression of cardiovascular stress.

### 2.4. Cardiac Events

In many studies, supraventricular extrasystoles (SVESs) and ventricular extrasystoles (VESs) are associated with adverse cardiovascular outcomes [11]. Therefore, SVESs and VESs per hour were defined as cardiac events. Cardiac events were reported for total time, standby time and rescue operation time.

### 2.5. Statistical Analysis

A total of 52 operational situations were included in the study. Due to incorrect measurements and artefacts, a certain number of data could not be included in the study evaluation. For the BP sys mean and BP dia mean, seven missions could not be considered; for BP sys min, BP dia min, BP sys max and BP dia min, nine missions could not be included; for HR mean, four operations were not evaluated; and for HR min and HR max, we excluded eight missions.

Investigators performed all statistical analysis using Stata/IC 16.1 for Unix (StataCorp LP, College Station, TX, USA) and IBM SPSS Statistics 25. Anthropometric parameters, 10-year risk and BP and HR characteristics were described using mean, standard deviation (SD), median, minimum and maximum values. Differences between standby time and during rescue operation were estimated using random-effects linear regression; 95% confidence intervals (CIs) were also reported. Cardiac events were defined as absolute values and 95% CIs. The event rate per hour are absolute values. All statistical tests were two-sided with a significance level of 0.05.

## 3. Results

### 3.1. Study Population

The mean age of all participants was 40.6 ± 7.7 years (31–59 years). The emergency physicians were 37.0 ± 3.0 years old; the paramedics were 44.6 ± 9.35 years old. The average height of the paramedics was 1.83 ± 0.07 m, and the average body weight was 84.2 ± 9.7 kg. The BMI corresponds to 24.9 ± 1.5 kg/m^2^. The emergency physicians were 1.82 ± 0.09 m in height, 81.9 ± 10.4 kg in body weight and 24.6 ± 2.6 kg/m^2^ in BMI. The average operational experience in air rescue was 7.0 ± 7.9 years (emergency physicians 2.9 ± 2.4 years, paramedics 11.4 ± 9.4 years). The 10-year cardiovascular risk of the emergency physicians was classified as low (low risk <5%). The highest 10-year risk was detected as 6% for one paramedic (emergency physicians 0.3 ± 0.5%, paramedics 1.0 ± 1.7%). Table 1 shows the anthropometric characteristics of the study group.

### 3.2. Medical History

Four participants reported high blood pressure with medical treatment as a pre-existing condition. All four received antihypertensive monotherapy with ACE inhibitor (one participant), AT1 receptor blocker (two participants) or calcium antagonist (one participant). Other cardiovascular and respiratory diseases were not known. One participant had the diagnosis of a herniated disc without neurological symptoms, and one other reported a meniscal tear in the left knee.

### 3.3. Operating Characteristics

HEMS crew members had an average of 2.7 ± 1.3 rescue operations per shift (total 52) with an average duration of 53 ± 34 min. Over the day, the mean total operating time totaled 158 ± 96 min. The distribution of operational reports was 26 (50%) internal emergencies, 17 (32.7%) traumatological and surgical emergencies, 5 (9.6%) resuscitations and 4 (7.7%) other emergencies.

### 3.4. Blood Pressure

**Figure 1:**
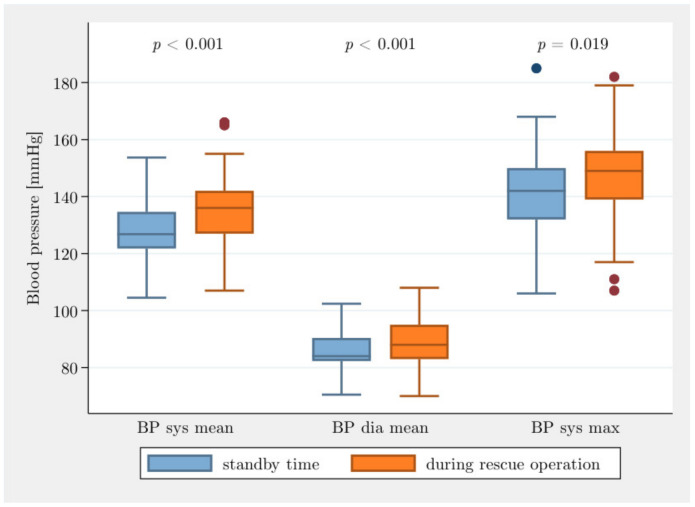
Blood pressure (BP sys mean, BP dia mean, BP sys max) during standby time and rescue operations.

Table 2 provides a descriptive overview of selected BP variables in standby time and rescue operation. The BP mean values in standby time were 127.6 ± 11.4 mmHg systolic BP and 85.3 ± 7.3 mmHg diastolic BP. During the period of rescue operations, the BP mean values were 135 ± 13.6 mmHg for systolic BP and 88.7 ± 9.1 mmHg diastolic BP. The difference in the mean BP sys mean was 7.4 ± 9.0 mmHg and was statistically significant (7.4, CI [5.1; 9.7], *p* < 0.001). The verification of the hypothesis that the stress during an operation is higher than during the rest period was carried out with a connected *t*-test of the BP measurements. The resting BP value was measured while sitting and compared to the BP sys mean during the first rescue operation of the working day. There was a significant difference between the two measured values (*p*-value < 0.001; CI [4; 20]). The individual values of the participants on standby time were lower than those obtained during the period of a rescue operation. During rescue operations, the average systolic BP values were on average 14.2 ± 11.4 mmHg higher than in standby time. The values of the BP dia mean (*p* < 0.001) and BP syst max (*p* = 0.019) were significantly higher during rescue operations than on standby time. The changes to BP syst mean, BP dia mean and BP sys max are shown in Figure 1.

### 3.5. Holter (24 h) ECG

The HR at maximum, minimum and average was measured for HEMS crew members during 48 rescue missions in the standby time and during the rescue operations (Table 3). In four cases, no data could be evaluated for HR min and HR max. None of the study participants experienced pathological arrhythmias or signs of ischemia during the measurements. Nevertheless, irregularities in cardiac activity were recorded in each subject. VESs occurred most frequently with an average of 163 VESs per day of use. Furthermore, on average 24 SVESs, four missing QRS complexes and two supraventricular tachycardias were recorded.

**Figure 2:**
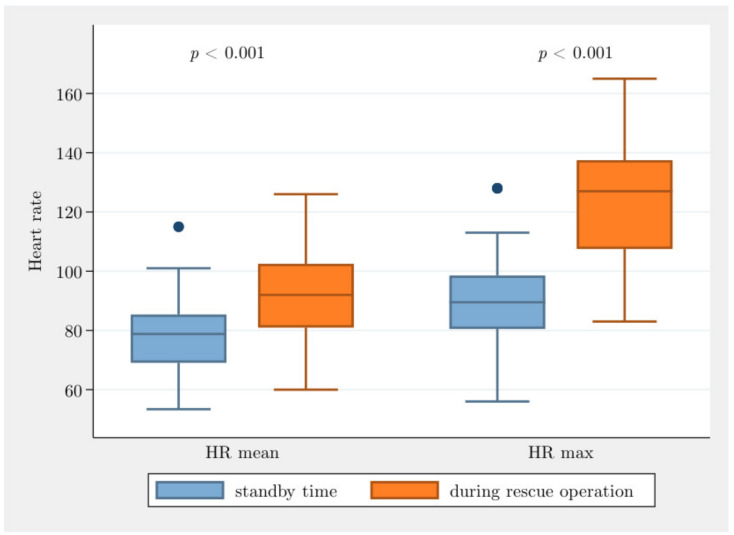
Overview of mean (HR mean) and maximum (HR max) heart rates during standby time and rescue operations.

Significant differences between the values in the standby time and during the operations time can be detected for the HR mean, HR max and HR min. The HR mean was on average 13 bpm higher during operation time than on standby time (13.0, CI [10.8; 15.3], *p* < 0.001). The HR max was on average 33.7 bpm higher during the operation time than in the standby time (33.5, CI [26.2; 40.8], *p* < 0.001), and the HR min was on average 7.2 bpm higher during the operation time than in the standby time (7.2, CI [5.1; 9.4], *p* < 0.001) (Figure 2).

### 3.6. Cardiac Event Rates

The measurements show a total of 2514 cardiac events during the standby period and 770 cardiac events during the rescue operation period. Cardiac events occurred significantly more frequently during the period of rescue operation than in standby time hours (*p* = 0.02). As shown in Table 4, the event rate outside of the operating time was 11 per hour (CI [10.6–11.5]), while the event rate in the rescue operation period was 16.7 per hour (CI [15.6–18.0]).

## 4. Discussion

The present study aimed to determine the stress on the cardiovascular system during rescue operations in helicopter emergency medical service. The cardiovascular load was determined by recording the BP and HR values and the derivation of the long-term ECG in rescue operations. These values were compared to values during standby time.

The working conditions in helicopter emergency medical service cause physical stress that can put the human organism in extreme stress situations [12]. HEMS crew members are exposed to the following stressors: shift and night work, unpredictable alarms resulting in abrupt stress on the cardiovascular system and physically and psychologically challenging medical situations during repetitive missions, as well as flight-related stress situations and unpredictable situations.

In the examined cohort, none of the participants showed systolic hypertension (BP sys > 140 mmHg), and 28.6% showed increased diastolic (BP dia > 90 mmHg) values at rest. The mean resting values of the HEMS crew members were at 123/83 mmHg in the normal range. The study collective showed a significantly lower average resting BP value compared to colleagues working in the ground-based emergency services. These showed systolic hypertension in 17.5% and diastolic hypertension in 40.2% [13]. Moreover, they were below the German national average, in which 31.8% of the German population was found to have high BP [14].

Except for the BP dia min, the examined cohort showed significantly higher BP values in rescue operations than during standby time. In regard to differences between BP values in rescue operations, the studied cohort showed significantly higher mean systolic BP values of 14.2 ± 11.4 mmHg. Overall, there was a significant increase in mean systolic BP of 7.4 ± 9 mmHg and diastolic BP of 3.4 ± 6 mmHg over the entire working day during rescue operations.

Up to now, there have hardly been any studies on changes in BP during air rescue operations. It is, therefore, difficult to assess the results we have collected due to the lack of comparable data. Investigations have been conducted in related occupational groups with a similar occupational load profile, namely the ground-based emergency services and the fire department. More than 20% of firefighters have hypertension [15]. Studies in this occupational group indicate that increased systolic BP is an independent predictor of coronary events on duty [16,17]. Arterial hypertension is considered to be one of several main risk factors that can be influenced by a cardiovascular load in the professional group of emergency services [18,19]. Kales et al. [20] were able to demonstrate that this is insufficiently controlled in the professional group of the rescue workers and, therefore, significantly increases the risk of cardiovascular events.

In the general population, cardiovascular events frequently occur in the morning [21]. This increase in time is justified by the fact that the cortisol level is higher in the morning, catecholamines are released, and BP and HR increase. Cardiovascular deaths during firefighters’ work hours, on the other hand, do not follow this circadian pattern but occur more frequently between noon and midnight [17]. This fact clarifies that there is a more significant influence through individual risk constellations and work-related response situations and may explain a significantly higher prevalence of cardiovascular risk factors in cardiovascular deaths [17]. The research group led by Kales et al., identifies professional and personal risk factors associated with cardiovascular deaths during firefighting duties. These are a deviating circadian rhythm in the context of shift work and the highest risk of death during extremely physically demanding activities. It can be assumed that these factors are relevant for HEMS crew members and contribute to an increased cardiovascular risk.

An Italian study by Carchietti et al. [8] examined the influence of stressors on the HEMS crew members during the operation by determining the BP and HR values before and after the flight. The resting BP values were slightly lower compared to our study (Carchietti et al.: systolic 120.2 ± 13.2 mmHg and diastolic 75.4 ± 11.1 mmHg vs. our study results: BP sys resting 123 ± 7.8 mmHg; BP dia resting 83 ± 7 mmHg).

Adams et al. [22] carried out a study with long-term BP measurements and long-term ECG recordings with physicans during 24-h hospital services and in their leisure time. The data indicate a mean BP increase during the service period, which is significant in the diastolic range. At 125.8/82.5 mmHg, this was higher in service than in leisure time (123.8/77 mmHg).

The mean resting HR was in the normal range with an average of 73 ± 9.7 bpm. The HR is an easy-to-measure parameter and can be used to document psychological and physical stress [23]. In 2007, the European Cardiology Society declared increased resting HR as an independent risk factor for cardiovascular diseases [24]. The background was the finding of an increased risk of cardiovascular morbidity and mortality with increased HR. Based on the Framingham study data, a long-term high cardiovascular risk with increased resting HR was confirmed, whereby no limit value was set [25]. Compared to the no-work time, the mean HR in our cohort was significantly higher at 91.1 ± 14.8 bpm than in the non-work time (mean HR: 78.1 ± 13.2 bpm), with an average difference of 13 bpm (CI [10.8; 15.3], *p* < 0.001). Carchietti et al. [8] also determined significantly increased HRs for HEMS crew members even after the end of the flight, with a difference of 3.9 ± 9.5 bpm. The reasons that can cause an increase in frequency in air rescue operations are diverse and include vibrations during the flight due to the rotors and psychological and physical stress from the rescue operation and noise [8,26].

All study participants had SVESs, VESs or both during the examination time.

Extrasystoles are widespread among healthy and clinically asymptomatic persons; SVESs occur in approximately 87% of healthy persons during long-term measurement and VESs occur in approximately 1% on resting ECG and in 40–75% of long-term measurements [27,28]. Frequent VESs (>60/h or 1/min) have an estimated prevalence of 1–4% in apparently healthy people and no worse prognosis compared to the normal population [29,30,31]. Recent clinical studies report that frequent VESs cause myocardial dysfunction [32,33]. Using the speckle tracking echocardiography technique, an ultrasound procedure for the objective representation of the myocardial function, Barutcu et al. [34] found decreased left ventricular cardiac function after VESs. An SVESs prevalence of 5–9% during the exercise test is known in healthy people, with an age-related increase in prevalence. VESs, however, were registered at a prevalence of 21–44% [35,36,37].

The comparison of the rescue phase time with the standby time in the present study showed a significantly more frequent occurrence of SVESs and VESs during the rescue operation phase. VESs and SVESs were significantly more frequent during the operational phase than during the standby time (16.7 vs. 11.0 events/h). This fact can be seen as an expression of increased cardiovascular stress.

Maurer et al. [38] were unable to establish an association between exercise-induced SVESs and cardiovascular mortality or coronary mortality in a follow-up in 1383 asymptomatic volunteers. The study by Morshedi-Meibodi et al. [39], as part of the Framingham Heart Study, showed that the occurrence of VESs in the stress test in asymptomatic people was associated with increased mortality. Age, male gender and hypertension were correlated in this cohort to stress-induced VESs. In the long-term observation, 60–80% of these were associated with an increased risk of mortality according to frequency. Data from the Paris Prospective Study were used to test whether stress-induced VESs are a physiological response to training [40]. The authors conclude that in middle-aged asymptomatic men, the occurrence of premature ventricular depolarizations during exercise is associated with a long-term increase in the risk of death from cardiovascular causes.

Smith et al. [41] examined a group of firefighters in the United States with no known medical history for ECG changes during a 12-h post-firefighting period and a control period. Researchers examined firefighters after a complex simulation firefighting that lasted about 30 min. SVESs and VESs, as well as ST segment changes that may indicate myocardial ischemia occurred. Earlier examinations provided indications for ECG changes due to a fire-fighting phase [42,43]. These could explain the high risk of sudden cardiac death in this occupational group [44,45]. A pilot study in New York analyzed long-term ECG of firefighters during duty and 16 h immediately afterward, and they detected in 57% (*n* = 28) high rates of VESs during observation time [43]. Carey et al. [43] described non-persistent ventricular tachycardias with ≥3 consecutive VESs in 11% of the test persons, without accumulation during the period of use, but with a significantly higher prevalence compared to the normal population.

## 5. Limitations

This study contains some points that limit the relevance of the results. The study was conceived as a pilot study; therefore, the study group is a small cohort. More extensive prospective studies with larger sample sizes are necessary to support or refute the results. Another limitation of the study is the lack of data collected on a completely free day. It was not possible to collect these data because of the stressful job schedule of the study participants. The control data in this study group were collected during the recovery time between rescue operations.

## 6. Conclusions

Increased resting blood pressure and resting heart rate values were not found more frequently in the HEMS crew members when compared to the general population. We assume that the cardiovascular risk of HEMS crew members corresponds to that of the average population. We recommend a lower risk profile in the background of the occupational burden. However, this study shows that recurring physical stress events occur during the rescue operation, which burdens the cardiovascular system. This stress burden explains the significantly increased occurrence of extrasystoles and the considerably higher heart rate and blood pressure values during the rescue operation. We assume that work stress has potentially harmful effects on individual health, especially in crew members with pre-existing cardiovascular risk factors, and that they can significantly increase the cardiovascular risk.

It is essential to carry out general prevention programs as part of continuous risk stratification of HEMS crew members to prevent cardiovascular events in active HEMS missions. Overall, up to date, the operational cardiovascular burden on air rescue personnel has been insufficiently studied. Firefighters often serve as an example, and there are numerous studies worldwide on recording cardiovascular risks in emergency work and approaches for targeted preventive measures. Based on these studies, preventive procedures should also implement in crew members of helicopter emergency service.

## Figures and Tables

**Table 1:** Basic characteristics and 10-year risk of the study group (all participants, emergency physicians, paramedics).

	All Participants				Emergency Physicians				Paramedics			
	*n*	Mean	SD	Median	*n*	Mean	SD	Median	*n*	Mean	SD	Median
Age (years)	21	40.6	7.7	38.0	11	37.0	3.0	36.0	10	44.6	9.35	44.0
Height (m)	21	1.83	0.08	1.83	11	1.82	0.09	1.82	10	1.83	0.07	1.83
Weight (kg)	21	83.0	9.9	84.0	11	81.9	10.4	82.5	10	84.2	9.7	84.2
BMI	21	24.8	2.1	25.1	11	24.6	2.6	24.9	10	24.9	1.5	25.2
Waist circumference (cm)	21	89.4	7.1	90.0	11	88.1	8.4	90.0	10	90.9	5.4	90.5
Professional experience (years)	21	7.0	7.9	4.0	11	2.9	2.4	3.0	10	11.4	9.4	8.0
10-year risk (%) (ESC score)	21	0.6	1.3	0	11	0.3	0.5	0	10	1	1.7	0.5

**Table 2:** Blood pressure values during rescue operations and in standby time.

		Description				Linear Regression **	
		Mean	SD	Median	Min–Max	Estimated Mean (95% CI)	*p*-Value
BP sys mean *	standby time	127.6	11.4	126.8	104.5–153.7	128.0 (123.6; 132.4)	
*n* = 45	rescue operation	135.0	13.6	136.0	107.0–166.0	135.6 (130.1; 141.1)	
	difference	7.4	9.0	7.2	−16.0 to 31.0	7.4 (5.1; 9.7)	<0.001
BP dia mean *	standby time	85.3	7.3	84.0	70.5–102.4	85.2 (82.4; 88.0)	
*n* = 45	rescue operation	88.7	9.1	88.0	70.0–108.0	89.1 (85.5; 92.9)	
	difference	3.4	6.0	3.5	−12.0 to 20.0	3.6 (1.8; 5.5)	<0.001
BP sys min *	standby time	114.7	12.6	114.0	84.0–143.0	114.6 (109.7; 119.4)	
*n* = 41	emergency operation	124.7	13.0	125.0	102.0–152.0	125.2 (119.5; 103.8)	
	difference	10.0	8.9	9.0	−8.0 to 28.0	10.1 (7.0; 13.3)	<0.001
BP dia min *	standby time	76.4	8.7	77.0	50.0–94.0	76.4 (73.0; 79.8)	
*n* = 41	rescue operation	82.8	9.5	84.0	63.0–104.0	83.1 (78.8; 87.5)	
	difference	6.4	7.0	7.0	−7.0 to 24.0	6.7 (3.8; 9.5)	<0.001
BP sys max *	standby time	141.8	15.5	142.0	106.0–185.0	142.4 (136.5; 148.4)	
*n* = 41	rescue operation	147.4	17.3	149.0	107.0–182.0	147.7 (141.9; 153.5)	
	difference	5.6	16.5	6.0	−42.0 to 48.0	5.6 (0.9; 10.3)	0.019
BP dia min *	standby time	95.7	10.5	94.0	78.0–124.0	95.8 (91.9; 99.7)	
*n* = 41	rescue operation	95.6	11.2	95.0	70.0–117.0	96.0 (91.6; 100.4)	
	difference	−0.1	11.4	2.0	−45.0 to 25.0	0.0 (−3.9; 3.9)	1.000

* Participants whose measurements were recorded before and during a rescue only; ** random-effects linear regression. BP = blood pressure, CI = confidence interval.

**Table 3:** Heart rate (HR max, HR min, HR mean) during rescue operations and in standby time.

		Description:				Linear Regression **	
		Mean	SD	Median	Min–Max	Estimated Mean (95% CI)	*p*-Value
HR mean *	standby time	78.1	13.2	78.8	56.4–115.0	76.3 (71.3; 81.6)	
*n* = 48	rescue operation	91.1	14.8	92.0	60.0–126.0	89.5 (83.8; 95.1)	
	difference	13.0	9.4	12.3	−11.0 to 37.5	13.0 (10.8; 15.3)	<0.001
HR min *	standby time	67.9	12.7	68.0	44.0–100.0	67.3 (61.9; 72.6)	
*n* = 44	rescue operation	75.1	14.1	74.0	49.0–111.0	74.6 (69.2; 80.0)	
	difference	7.2	9.1	6.0	−9.0 to 33.0	7.2 (5.1; 9.4)	<0.001
HR max *	standby time	90.0	15.4	89.5	56.0–128.0	89.8 (84.5; 95.1)	
*n* = 44	rescue operation	123.7	21.0	127.0	83.0–165.0	122.9 (115.1; 130.8)	
	difference	33.7	20.5	36.0	−18 to 70.0	33.5 (26.2; 40.8)	<0.001

* Participants whose measurements were recorded before and during a rescue only; ** random-effects linear regression.

**Table 4:** Cardiac event rates (total, standby time, rescue operation).

	Time (Hours)	Cardiac Events	Event Rate Per Hour	95% CI *	*p*-Value **
Standby time	227.8	2514	11.0	10.6–11.5	
Rescue operation	46.0	770	16.7	15.6–18.0	0.020
Total	273.8	3284	12.0	11.6–12.4	

* Assuming Poisson-distributed number of events; ** random-effects Poisson regression.

## Data Availability

The datasets analyzed during the current study are available from the corresponding author on reasonable request.

## Abbreviations

BMI	Body mass index
ECG	Electrocardiography
ESC	European Society of Cardiology
HEMS	Helicopter emergency medical service
HR	Heart rate
HR max	Maximum heart rate
HR mean	Average heart rate
HR min	Minimal heart rate
BP	Blood pressure
BP dia max	Diastolic maximum blood pressure
BP dia mean	Diastolic average blood pressure
BP dia min	Diastolic minimum blood pressure
BP sys max	Systolic maximum blood pressure
BP sys mean	Systolic average blood pressure
BP sys min	Systolic minimum blood pressure
SVES	Supraventricular extrasystole
VES	Ventricular extrasystole
